# The Body’s Cellular and Molecular Response to Protein-Coated Medical Device Implants: A Review Focused on Fibronectin and BMP Proteins

**DOI:** 10.3390/ijms21228853

**Published:** 2020-11-23

**Authors:** Yi-Fan Chen, Clyde Goodheart, Diego Rua

**Affiliations:** 1 Center for Devices and Radiological Health, Food and Drug Administration, Silver Spring, MD 20993, USA; yi-fan.chen@fda.hhs.gov; 2 Celigenex, Inc., Fort Lauderdale, FL 33308, USA; clyde@celigenex.com

**Keywords:** hip, shoulder, implants, FN, protein, cytokines, neutrophils, macrophages, osteogenesis, titanium

## Abstract

Recent years have seen a marked rise in implantation into the body of a great variety of devices: hip, knee, and shoulder replacements, pacemakers, meshes, glucose sensors, and many others. Cochlear and retinal implants are being developed to restore hearing and sight. After surgery to implant a device, adjacent cells interact with the implant and release molecular signals that result in attraction, infiltration of the tissue, and attachment to the implant of various cell types including monocytes, macrophages, and platelets. These cells release additional signaling molecules (chemokines and cytokines) that recruit tissue repair cells to the device site. Some implants fail and require additional revision surgery that is traumatic for the patient and expensive for the payer. This review examines the literature for evidence to support the possibility that fibronectins and BMPs could be coated on the implants as part of the manufacturing process so that the proteins could be released into the tissue surrounding the implant and improve the rate of successful implantation.

## 1. Introduction

### 1.1. Background

Recent years have seen a marked rise in implantation into the body of a great variety of devices: hip, knee, and shoulder replacements, pacemakers, meshes, glucose sensors, and many others. Cochlear and retinal implants are being developed to restore hearing and sight. After surgery to implant a device, a layer of endogenous proteins adsorbs to and coats the surface of the device (unless it is already protein-coated). This coating results in attraction, infiltration, and attachment of various cell types including monocytes, macrophages, and platelets. These cells release chemokines and cytokines that recruit tissue repair cells to the device site. The adherent proteins interfere with the function of some devices, such as glucose sensors, while in other cases the proteins and the cells they attract lock the implant in place and are therefore beneficial.

The number of devices currently being used, and the expected increase in coming years, together with the many variations including different metals and alloys, different plastics, different coatings, and different surface finishes on the devices, results in a huge number of combinations.

In 2007, it was reported that total hip arthroplasty was a highly successful procedure but about 10% to 18% of the replacements required revision surgery [1], while a report in 2008 found that 68% of revisions were due to aseptic loosening or instability [2]. According to the Swedish Total Hip Replacement Registry [3], the rate of revision surgery in that country has decreased from about 15% to about 10% from 2000 to 2018. Although at about 5% or less, revision surgery for knee implants is also a significant problem [4].

### 1.2. Objective

Our sense of a need to consider recent insights on medical implants, together with previous experience (by CG) with a trial to attempt to grow new bone around teeth loosened by periodontal disease using hydroxyapatite and fibronectin, provided the impetus for this literature review [5]. Here, we focus on one metal, titanium, and review the protein coatings that can be applied to influence the cellular response to the implant when it is used as a joint replacement. The goal is to stimulate debate and advancement towards improving the success rate of implants for the benefit of patients as well as the health care system.

## 2. Cellular and Molecular Response to Implanted Medical Devices

### 2.1. Wound Healing

Implanting a device usually requires inflicting a wound, which starts the wound healing process. This process normally proceeds through a series of well-orchestrated stages that overlap each other in time. Hemostasis begins almost immediately. Blood coagulation with rupture of platelets seals small vessels and stops or slows bleeding. Clots are formed by plasma fibronectin (pFN) interacting with fibrin, which is converted from fibrinogen. Contents from ruptured platelets, which are released into the wound, include platelet-derived growth factor (PDGF), transforming growth factors (TGFs), and vascular endothelial growth factor (VEGF) [6].

The various signaling factors released into the wound by platelets usher in the second, inflammatory, phase that begins within hours of wounding and may last two weeks or more. Cleanup of the wound occurs during this phase, starting with the invasion of neutrophils, which begin phagocytizing debris and bacteria. Neutrophils also secrete various substances causing the classical signs of inflammation in and around the wound. Later, macrophages enter the wound. They also phagocytize debris and secrete growth factors. Lymphocytes follow and may start an immune reaction [6].

PDGF released by platelets during clotting attracts fibroblasts into the wound, initiating the proliferative phase. Fibroblasts begin producing collagen and other structural proteins; some fibroblasts convert to myofibroblasts containing actin. These cells help pull the wound together. Angiogenesis also begins in the proliferative phase, stimulated by VEGF and hypoxia in the wound [6].

Remodeling of the wound can take up to a year, during which collagen that was originally laid down randomly in the wound is removed and replaced with a more orderly arrangement of the collagen fibers. Blood vessels may be reconstructed improving blood supply in the wounded area [6].

More detailed descriptions of the phases of normal wound healing can be found in many publications, including [6]. But wounds do not always heal according to the normal progression. A wound that fails to heal within a three-month period is said to be a chronic wound. Failure to heal can occur if the wound is infected or if the patient has diabetes, venous stasis, or continuous pressure on the skin that impairs blood flow to the area [7]. Chronic wounds are characterized by excessive amounts of proteases in the wound bed. Proteases degrade proteins that are involved in healing, including fibronectin [8]. It is interesting to hypothesize that implant failure may be due to excess production of proteases in the immediate environment of the implant, mimicking a chronic wound, in relation to macrophage function as will be discussed later in this review.

### 2.2. Bone Healing

Bone healing or regeneration is a highly complicated process, regulated by many factors including the complex composition of the tissue, microenvironment around the bone, the composition of extracellular matrix (ECM) insoluble proteins and glycoproteins, and cell-to-cell and cell-to ECM interactions. Based on the knowledge of this intricate process, various modifications of bioengineering implants have been developed for mimicking a pro-regenerative microenvironment around implants. To develop osteoinductive implants, bio-functionalization of the surface coating to reproduce an optimal microenvironment for scaffolding and osteogenesis is vital for the performance of implants. Bone morphogenetic proteins (BMPs) stimulate osteogenesis; both the local concentration and distribution of BMPs and their isomer variations have a great influence on the performance of the biomaterials [9,10].

After an injury, including surgical placement of an implant, it is vital to have an appropriately activated healing mechanism. Bone healing sequential events include inflammation, mineralization, and bone remodeling. These events involve a broad crosstalk with the immune system through the regulation of immune cells, cytokines, growth factors, and molecular signaling pathways. Upon injury, the coagulation process generates a transient fibrin:fibronectin clot that serves as a matrix for recruiting immune cells, polymorphonuclear leukocytes (PMNs), monocytes, and tissue-resided macrophages. These cells initiate the acute inflammatory response [11]. Activated PMNs release proteolytic enzymes and pro-inflammatory cytokines that promote tissue remodeling and recruit myeloid cells and mesenchymal stromal cells (MSC), respectively [12].

Macrophages receive various cues from the surrounding environment and exhibit different phenotypes in the healing process. In the early stage of healing, macrophages polarize toward the M1 phenotype and release inflammatory cytokines, recruit immune cells, and eradicate dead cells and debris. In the late stage, macrophages are alternatively activated into the M2 phenotype that releases tissue repair factors and anti-inflammatory cytokines, such as IL-10, TGF-β, and VEGF. These factors suppress inflammation and facilitate endochondral bone formation [13]. Monocytes and tissue-resident macrophages essentially participate in bone repair and remodeling; depletion of osteo-macrophages can result in complete loss of osteoblast bone-forming surface in vivo that suggest disruption of endosteal niches [14,15].

Owing to the vital immunomodulatory characteristics of endogenous ECM components, more and more studies look into engineering ECM or biomaterials for mediating an appropriate transition of macrophage polarization during wound healing to favor bone regeneration by endochondral ossification [13,16,17,18]. Bone morphogenetic proteins (BMPs) are involved in the initial stages of endochondral ossification. Recombinant human BMP-2 (rhBMP-2) has been applied in numerous studies for bone-healing medical devices and biomaterials [19,20,21,22]. Macrophages can initiate osteoinductive signals to induce BMP-2 expression that indicates their capacity in regulating osteoinduction during the bone healing process [23]. Therefore, it is important to consider immunoregulatory features against macrophages when using ECM components or BMPs in the development of biomaterials.

Ideally, biocompatible materials should be inert and initiate little or no damage to surrounding tissues such as bone. However, biomaterials that have contact with tissues may evoke a host immune response, and the ability to resolve the initial inflammation determines the proficiency of biointegration of the implanted biomaterials as will be explained in the following subsections.

### 2.3. Immune Response

Implantation of a bio-coated medical device may induce a host immune response. This response could adversely affect the integration and performance of the implant. Despite the improvement of biomaterial engineering over the past two decades, adverse immune reactions such as excessive inflammation, impairment of healing, fibrotic encapsulation, tissue destruction, and isolation or rejection of a medical device, are still the main hurdle for long-term sustainability of implants. Therefore, regulating appropriate immune responses at an implantation site is one essential consideration in designing biomaterials. Macrophages play critical roles in both innate and adaptive immune systems that respond against foreign bodies and may participate in pro-regenerative remodeling.

Understanding the crosstalk between the skeletal and immune systems in the bone remodeling process, known as osteoimmunology, is important when developing bone grafts and bone-associated biomaterials [24]. Excessive immune response after implantation is still one of the major factors hampering long-term survival and function of biomaterials.

### 2.4. Foreign Body Reaction

The functionality of an implant can also be compromised by a persistent foreign body reaction, which happens when there is prolonged inflammation characterized by fibrotic encapsulation, macrophage infiltration, and foreign body giant cells from fused macrophages [25].

## 3. Biological Coatings on Medical Devices

### 3.1. Biological Coatings Used or Investigated on Some Medical Devices

We searched for use of BMPs and fibronectin as components of medical devices. Some examples from searches of published literature, relevant public FDA databases—as well as clinical trials.gov are shown in Table 1. The devices found revolve around dental and orthopedic indications of use.

### 3.2. Fibronectin: A Cell-Adhesive Protein

ECM-derived proteins contain adhesive motifs that promote cell adhesion and spreading. Fibronectin is an ECM component that plays a key role in mediating cells’ adhesion, growth, migration, and differentiation during bone remodeling. Especially in bone regeneration, fibronectin is involved in bone formation and osteoblast differentiation [30,31]. Fibronectins are cell-adhesive ECM glycoproteins that exist as soluble forms in plasma and less-soluble cellular forms to be assembled into the ECM [32]. ECM cell-binding proteins, i.e., collagen, fibronectin, vitronectin, and laminin, can induce in vitro osteogenic differentiation of mesenchymal stem cells. Further, fibronectin and laminin support stretching-induced osteogenic differentiation better than collagen and vitronectin do [33,34].

Possible strategies to enrich fibronectin deposition on the implanted site include direct adsorption of fibronectin on the implant surface or enhanced fibronectin retention by a layer on the implant of fibronectin-binding or attractive substrates (Table 2). Another way to promote cell adhesion to achieve ECM-like functionality in binding to cell surface integrins is to use recombinant protein fragments and short polypeptides, i.e., fibronectin-derived adhesion motifs, such as RGD, PHSRN, REDV, and LDV, which contain the integrin-binding domain for fibronectin.

### 3.3. Enhancing Fibronectin Surface Binding

Owing to the essential role of ECM proteins in cell adhesion, the direct application of ECM proteins to a biomaterial surface was introduced in the early era of biomaterial development. ECM-derived proteins, especially fibronectin, were investigated broadly in biomaterial surface coatings to facilitate cell adhesion and proliferation.

Protein adsorption onto an implant surface can also happen shortly after implantation by the recipient’s body, in a non-specific manner. The deposition of proteins on a surface is under the influence of their affinity for the surface, and on their size and concentration in the surrounding tissue microenvironment. Therefore, controlling the amount, composition, and conformation of protein adsorption is key to the surface compatibility of well-designed biomaterials [35]. In the covalent immobilized grafting of fibronectin, the surface conjugation scheme includes the type of crosslinks, surface topography, and roughness, which affect the protein conformation and subsequent bioactivity [37]. Lin et al. reported that the fibronectin adsorption force on self-assembled monolayers followed a chemistry dependence of -NH_2_ > -CH_3_ >> -OH, as well as demonstrated that the fibronectin adsorption force also regulates late osteoblast adhesion and fibrillogenesis of endogenous fibronectin [36]. These findings provide insights for selecting surface chemistry or conjugation strategy for future implant development.

Immobilization of monoclonal antibody has been investigated for use on implant surfaces. However, monoclonal antibodies have numerous limitations, such as immunogenicity, low stability, and high production cost [55,56].

Aptamers can also be used to increase the selective binding and retention of fibronectin on an implant surface from extracellular space [39]. Aptamers are small oligonucleotides that recognize and bind proteins with high affinity and specificity through their three-dimensional, highly specific conformation [57]. Aptamers have low immunogenicity and low toxicity, they are able to bind to smaller ligands, they are not directly recognized by the human immune system as foreign agents, and their modification and production are more cost-efficient than antibodies [58]. Galli et al. proposed using ssDNA aptamers against fibronectin in a thiolate hyaluronic acid/diacrylate polyethylene glycol hydrogel (tHA/PEGDA) to promote the attachment and growth of osteoblastic cells [38]. This functionalized hydrogel was enriched with aptamers that selectively bind to fibronectin, which increased the number of adherent human osteoblasts and presented a more spreading adhesion [38]. The same research group made another study, this time using a chitosan film. The aptamer-enriched chitosan promoted cell proliferation of murine MC3T3-E1 osteoblast cells in a dose-dependent manner [39]. Aptamers preserve an advantageous geometrical conformation of fibronectin for cells and thus regulate proper cell activation and proliferation, although the amount of fibronectin adsorption is not proportional to the number of coated aptamers [33,39,40].

### 3.4. Immobilizing Integrin-Binding Peptides on an Implant Surface

Fibronectins are large macromolecules with only a few short peptide sequences that serve as integrin-binding domains and that trigger cell adhesion and signaling. Using the full-length fibronectin polymer is limited because it is costly to produce in large quantities. It is also difficult to modify, characterize, and control its conformation on a biomaterial surface. In contrast, ECM-derived fragments or binding domain-mimicking peptides can be synthesized or expressed in large quantities, immobilized on implant surfaces at high densities, and modified for specific applications. The cell-binding peptide sequences in fibronectin are RGD, PHSRN, REDV, and LDV [41,45,46,47]. The coating density and the stability of these peptide ligands on the surface of implants influence implant performance.

RGD is an adhesive peptide sequence in many ECM proteins, such as fibronectin and vitronectin. Fibronectin contains the RGD sequence and PHSRN site, and the presence of PHSRN synergistically increases RGD’s affinity to α5β1 integrin [41,44]. The colocalized synthetic oligopeptide of RGD and its synergistic PHSRN motifs in a poly(ethyl glycol) hydrogel improved osteoblast adhesion, spreading, and focal contact formation [43]. The co-assembly of two self-assembling peptides, RGD, and PHSRN, that produced a self-supporting hydrogel also exhibits synergistic effects in adhesion, spreading, and proliferation in vitro compared to RGD alone [42]. Further, the finish of an implant surface, whether it is smooth and polished or rough, is important for RGD coating effectiveness at osteointegration.

FNIII7-10 is an engineered recombinant fragment of fibronectin that includes the 7–10th type III repeats of fibronectin and binds specifically to the α5β1-integrin [48]. A FNIII7-10 functionalized titanium implant showed improved osseointegration and enhanced healing responses in vivo compared to RGD-functionalized titanium [52,53]. The structural stability of FNIII9-10 was reported to affect its integrin specificity and the downstream osteoblastic differentiation potential of mesenchymal stem cells (MSC) [49,50,54]. The recombinant FNIII9-10 and the structurally stabilized FNIII9*-10 (Leu1408 to Pro) variant demonstrated a higher MSC differentiation capacity than full-length fibronectin [50]. Engineered multifunction fibronectin recombinant that comprises FNIII9-10, the integrin-binding domain, and FNIII12-14 (the growth factor-binding domain), showed promise in wound and bone tissue healing [51].

### 3.5. Soluble Molecules: BMPs

Soluble biomolecules, such as growth factors, are essential in the process of bone regeneration. They facilitate MSC recruitment, tissue remodeling, and osteoblastic lineage proliferation upon bone injury. BMPs are multifunctional growth factors that promote osteogenesis and neovascularization of bone tissue. In bone tissue, BMPs are secreted from osteoprogenitor cells, osteoblasts, chondrocytes, and endothelial cells, and then stored in the ECM and released in bone repair and remodeling. BMPs are members of the TGF-β superfamily; more than 20 homodimeric or heterodimeric BMPs are known, and at least 7 of them have osteoinductive potential [59]. BMPs can be sorted into several subgroups according to their sequences and functions, such as BMP-2/-4 group and OP-1 group (osteogenic protein-1; BMP-5 to -8) [60,61]. BMP-2, -4, and -7 display osteogenic potential. Their heterodimers, such as BMP-2/6, BMP-2/7, and BMP-4/7, exhibit higher affinities for BMP receptors and osteoinductive capacity than most homodimers both in vitro and in vivo [62,63,64]. The heterodimers are proposed to initiate a combination of receptor signaling from different BMP subgroups that may contribute to a higher osteoinductive effect than homodimers [65,66,67].

BMPs can be purified from demineralized bone extracts to investigate their osteogenic potential [68,69]; however, bone extraction in a large quantity for therapeutic applications is cost-inefficient and brings concerns of batch-to-batch variation and immunogenicity. The maturation of cloning and recombinant systems ensures efficient and reliable expression of recombinant human BMPs (rhBMPs). Currently, heterodimers are the most commonly used forms of BMPs for investigating their potential in bioengineering [61]. Owing to their osteoinductive activity, BMPs are adsorbed to biomaterials to investigate their potential in bone tissue regeneration; however, the side effects, such as ectopic bone growth, inflammation, and seroma, set back the application of BMP biomaterials [70]. Many clinical trials have been designed to investigate the therapeutic potential of various rhBMP medical devices. Improving how rhBMP is delivered can help in alleviating BMP-associated side effects.

### 3.6. BMP Delivery Systems

Ideally, a BMP delivery system requires controlled release and sustained bioactivity at the implantation site. The system should avoid release burst and lateral diffusion, which can activate osteoclasts and subsequent bone resorption, and mineral deposition in muscle tissues, respectively [71,72]. Tissue engineering depends on three elements, biomaterial scaffolds, cells, and signaling, working together. BMP activates osteoinductive signaling; therefore, an appropriate osteoconductive scaffold or carrier is essential to make an effective BMP biomaterial. BMP carriers (Table 3) should be biocompatible, osteoconductive, concomitantly biodegradable with bone formation, and able to maintain an optimal concentration of BMP during bone regeneration and localize at the implantation site [73,74]. Collagen is the original bone component that is osteoconductive and supports mineralization; therefore, demineralized bone matrix and absorbable collagen sponges are used to entrap endogenous BMPs or to carry rhBMPs [75,76,77]. Other natural polymers, such as hyaluronic acid, alginate, gelatin, and chitosan, which provide scaffolding for cell adhesion and differentiation, also apply as BMP delivery systems [78,79,80,81,82,83]. In order to have a more controllable and tunable carrier system, synthetic polymers, like hydrogel, polylactic acid (PLA), and polyglycolic acid (PLG), are being evaluated for their use in delivering rhBMPs [84,85,86]. Small molecules or modifications can be introduced to enhance the immobilization of rhBMPs, through heparinization, succinylation, alkylation, addition of chondroitin sulfate, and covalent conjugation [87,88,89,90,91,92].

### 3.7. Fibronectin/ECM and BMPs as Combined Biomaterials

According to in vitro studies, BMPs act at small dosages: 5 ng to 20 ng per mL. But because of rapid proteolysis and clearance upon implantation, most commercially available BMP products and trial-testing products are made in supraphysiological doses [98,99]. High dosage of growth factor causes severe biomaterial-induced off-target side effects and it is costly to embed a large amount of recombinant proteins. Therefore, the challenge of designing BMP carriers is to improve their properties and functionalities without increasing their dosage. In material engineering, the integrin receptor signaling for stem cell adhesion can be controlled through modifications in surface chemistry. However, these chemically modified biomaterials usually have no concomitant control for growth factor binding and activity.

Bone regeneration is a complicated process, and the isolated action of one influential factor may not have enough effectiveness without interaction with other growth factors and cytokines. Therefore, the combined use of ECM proteins, such as fibronectin, with BMPs may allow a better presentation of growth factors and localize their bioactivity, and it may also provide a platform to study BMP biomaterial-mediated signaling and cell migration [95]. As mentioned in a previous section, engineered fibronectin recombinant fragments contain both cell adhesion domains and growth factor sequestering domains. These domains can combine a fibrin matrix with BMP-2 that promotes bone tissue repair [51].

Fibronectin adsorption onto a poly(ethyl acrylate)-coated surface can trigger spontaneous organization into a fibrillar fibronectin network that effectively presents BMP-2 and offers synergistic integrin/BMP-2 signaling [96,97]. This design of biomaterials achieves osteoblastic differentiation of mesenchymal stem cells in vitro and nonhealing bone defects repair in vivo with an approximately 300-fold lower BMP-2 concentration than using a collagen-sponge carrier [96]. Similar synergistic strategies are also being investigated using various BMP members co-coating with fibronectin onto medical implants. Brigaud et al. reported that fibronectin also acts synergistically with BMP-6 or BMP-7 on hydroxyapatite coatings; the affinity of a fibronectin-BMP complex for hydroxyapatite-coated titanium is BMP-6 > BMP-2 > BMP-7 [93]. Ettelt et al. used a biotin-streptavidin multilayer system to immobilize BMP-2/6 heterodimer with fibronectin. The system prevents the side effect from growth factor over-dosage and demonstrates enhanced selective osteoblast proliferation [94].

## 4. Immunomodulatory Biomaterials: Interplay with Macrophages

Upon implantation, M1 macrophages initiate the inflammatory response at the wound site, stimulate the angiogenic process, and secrete pro-inflammatory cytokines. A prolonged M1 existence results in foreign body responses that manifest fibrous encapsulation, granuloma, and chronic inflammation, and that eventually may result in failed biomaterial integration. M2 macrophages are alternatively activated macrophages that express anti-inflammatory cytokines and include different subsets based on their various functions in wound healing and immunoregulation [100,101]. M2c macrophages are the IL-10-stimulated M2 subset that is responsible for immune-suppression of inflammation, tissue remodeling, and promoting ECM synthesis by secreting growth factors and cytokines [102,103]. Unbalanced polarization of M1 and M2 may cause prolonged and excessive inflammation that compromises the healing process. The different states of macrophage polarization in the microenvironment at the implanted site can be tuned by biomaterials that shift to constructive remodeling, and thus minimize the inflammatory reaction and achieve a better healing outcome. A high M2 to M1 ratio around the implanted site was thought to be beneficial in remodeling; however, prolonged existence of macrophages after implantation can lead to the formation of foreign body giant cells [25,104,105]. Therefore, the challenge to optimize the use of biomaterials may rest on tuning their immunomodulatory function toward positive tissue remodeling and regeneration and enhanced biointegration.

### 4.1. ECM Scaffold Influences Macrophage Polarization

In the context of biomaterial implantation, cells receive a diverse range of biochemical or biophysical signals or both from the surrounding microenvironment and the implant. Implanted ECM biomaterials initiate the innate immune response that leads to a transient neutrophil accumulation followed by a sustained accumulation of macrophages through adhesion to the ECM. Mammalian source ECM has shown constructive remodeling outcomes in many studies [106,107]. However, solubilized ECM bioscaffolds derived from different tissues may influence the macrophage phenotype and subsequent remodeling outcome differently [107]. Additional research has shown that it is challenging to predict ECM-elicited macrophage responses without considering the source of the macrophages and ECM [17].

Researchers have proposed using ECM scaffolds with the sequential release of interferon-gamma (INFγ), the pro-inflammatory cytokine, and interleukin-4 (IL-4), the anti-inflammatory cytokine, for promoting M1 and M2 phenotypes consecutively. To achieve this design, Spiller et al. used the interaction differences between direct adsorption of INFγ and biotin-streptavidin conjugation of IL-4 to temper the release of cytokines and promote sequential M1 and M2 polarization [105]. Chen et al. developed a system of double hydrogel layers on titania nanotubes to allow rapid release of INFγ and sustained release of IL-4 that reaches M1 to M2 sequential action [108]. The same research group extended this design to bone implants to modulate a smooth transformation of M1 to M2 that may be helpful to the development of bone substitute materials [109]. Therefore, utilizing ECM-mimicking biomaterials may allow a more controllable platform to harness cytokines for regulating macrophage polarization in novel biomaterial development, thus leading to better healing and long-term stability of implants.

### 4.2. Fibronectin Regulates Macrophage Function

As previously described, fibronectin is a representative component of the ECM and has been developed in different forms of application in biomaterials. The biomimetic oligopeptide of fibronectin, RGD, is known for its role in modulating macrophage adhesion; however, the synergistic effect of presenting PHSRN together with RGD is reported to be associated with increasing macrophage fusion to form foreign body giant cells [110]. In a multiple sclerosis study, macrophages co-cultured with an aggregated fibronectin coating exhibit both pro-inflammation and anti-inflammation features [111]. Although the presentation of fibronectin aggregates in multiple sclerosis is pathological, topography of the fibronectin matrix on a biomaterial surface may alter macrophage performance. It is proposed that increasing surface roughness of implants may enhance fibronectin deposition and thus improve macrophage adhesion around the implanted site [112,113]. Surface hydrophilicity modulates macrophage polarization by regulating fibronectin adsorption and conformation, and subsequently alters behaviors of macrophages into anti-inflammatory and osteogenesis-promoting [114].

The colony-stimulating factor-1 receptor (CSF-1R) signaling regulates the proliferation, differentiation, and motility of macrophages [115]. The interaction between macrophages and fibronectin is vital for the host innate immune response and wound healing. A study has shown that the crosstalk between fibronectin-elicited src family kinases/focal adhesion kinase (SFK/FAK) signaling and CSF-1R in macrophages mediates fibronectin-induced migration of macrophages [116]. Thus, pharmacological modification of this crosstalk may be a strategy to adjust macrophage behavior during wound healing.

### 4.3. Immunomodulatory Role of BMP-2

BMP-2 is known for its role in osteoinduction for pluripotent mesenchymal stem cells; however, little is known about its effects in regulating macrophage activity and the subsequent osteoimmunoregulation in bone healing. An in vitro study showed that BMP-2 treatment can result in macrophage phenotypic switching from fully polarized M1 to M2-like. It also showed that BMP-2/macrophage-derived conditioned medium induces favorable osteogenic differentiation in bone marrow mesenchymal stromal cells [117]. In their study, the authors hypothesized that BMP-2 mediates immunoregulation through macrophage polarization. However, another study presents evidence that BMP-2 itself would not alter the macrophage phenotype, nor augment pro- or anti-inflammatory status in M1 or M2 macrophages, respectively [118]. Therefore, the influence of BMP-2 on macrophages and the immune environment around an implanted site needs further investigation.

## 5. Discussion

The vast majority of joint replacements do not fail or require revision surgery. Nevertheless, it is desirable to reduce the need for revisions. This review critically examined the role of biological materials that interact with the implants to identify possible approaches based on advances in knowledge of the biological responses to implants and wound healing.

The body’s response to a titanium implant is complex. Huo and Yue [119] reviewed many of the studies describing various coatings and surface treatments of titanium and its alloys, with a main focus on physical and chemical treatments. In many respects, the response resembles wound healing, because placement of the implant involves producing a wound. The body also sees the implant as a foreign body, which it certainly is, and it may respond accordingly. The body also may react immunologically to the foreign material in the implant. The ideal goal, however, is for the body to incorporate the implant into the bony structure in which it is placed so that the implant can perform as well as the joint that it is replacing.

Approaches include device surface functionalization with proteins or plasma-assisted surface coating after implantation, with the purpose of creating scaffolds for cell growth and eventual wound healing tissue integration. In addition to enhancing the integration of an implant into the diverse cellular tissues surrounding it, favorable host protein adsorption to implanted devices would reduce the threat of bacterial contamination. Further, implants would be expected to exhibit increased macrophage attachment and a concomitant increase of M2 macrophage presence that would reduce the likelihood of prolonged inflammation around the implant (i.e., improve tissue formation and wound healing).

Among the many proteins, cytokines, and growth factors that take part in the reaction to an implant, the various BMPs, and the fibronectins and their components stand out as being key players in the response.

The many approaches to modifying implants made of titanium and its alloys have shown a variety of cellular responses. The treatments include various chemical and physical alterations of the surface, the application of biological substances, and almost infinite combinations of surface treatment and coating. The different treatments and coatings have different responses, which opens the way for more personalized medical care. It may be possible to determine the best combination of surface treatment of the implant device and biological coating to optimize the benefit to each patient. “Smart” coatings of implant devices can be expected to one day improve the outcomes for patients.

## Figures and Tables

**Table 1:** Examples of biological coatings on implantable medical devices.

Medical Specialty	Type of Device	Biological Coating	Claims/Indication	Carrier/Vehicle	Reference/Clinical Trial
Dental	Bone grafting material	rhBMP-2 *	Induces new bone tissue at the site of implantation	Absorbable collagen sponge	[26]
Dental	Periodontal treatment	cFibronectin	Treat periodontitis including bone loss		[5]
Dental	Dental Implants	**rhBMP-2**	Bone Inductive Implant; Alveolar Ridge Abnormality		[27]
Orthopedic	Filler	**rhBMP-2**	Osteoinduction	Collagen scaffold with metal prosthesis	[28]
Orthopedic	Filler	**BMP-7/OP-1**	Osteoinduction, Degenerative Disc Disease	Collagen scaffold with metal prosthesis	[29]

rhBMP-2 = recombinant human BMP-2; cFibronectin = cellular fibronectin; OP-1 = osteogenic protein-1; * FDA-cleared/approved products are shown in bold type.

**Table 2:** Fibronectin-enhanced biomaterials.

Type	Biological Effects	Reference
**Increase pFN Adsorption**		
Self-assembled monolayers	Preferential covalent FN immobilization: -NH_2 _> -CH_3 _ >> -OH.	[35,36,37]
Aptamer	Oligonucleotides against fibronectin to promote cell adhesion.	[38,39,40]
**Increase integrin adhesion**		
FN polypeptides	Enhanced cell adhesion by adhesive peptides, e.g., RGD, PHSRN.	[41,42,43,44,45,46,47]
FN recombinant fragment	Enhanced cell adhesion and/or downstream osteoblastic differentiation by integrin-specific binding domains, e.g., FNIII7-10, FNIII9-10.	[48,49,50,51,52,53,54]

**Table 3:** BMP biomaterials.

Delivery Systems	Types	Reference
**Carriers**		
Natural Polymer	Collagen, demineralized bone matrix, hyaluronic acid, alginate, gelatin, and chitosan	[73,74,75,76,77,78,79,80,81,82,83]
Synthetic Polymer	Hydrogel, polylactic acid, and polyglycolic acid	[84,85,86]
**Carrier molecular modification**	Heparinization, succinylation, alkylation, and addition of chondroitin sulfate	[87,88,89,90,91,92]
**FN-adsorbing surface coatings**	Poly(ethyl acrylate), hydroxyapatite, and biotin-streptavidin multilayer system	[93,94,95,96,97]

## Abbreviations

BMP	bone morphogenetic protein
rhBMP-2	recombinant human BMP-2
FDA	U.S. Food and Drug Administration
